# The Effect of Obesity on Operative Time and Postoperative Complications for Peritrochanteric Femur Fractures

**DOI:** 10.7759/cureus.11720

**Published:** 2020-11-26

**Authors:** Hayden B Schuette, William M Durkin, Braden J Passias, Daniel DeGenova, Carina Bertolini, Philip Myers, Benjamin C Taylor

**Affiliations:** 1 Orthopedic Surgery, OhioHealth, Columbus, USA; 2 Orthopedics, Aultman Hospital, Canton, USA; 3 Orthopedic Trauma, OhioHealth, Columbus, USA; 4 Orthopedics, Singing River Hospital, Gulfport, USA

**Keywords:** obesity, hip fracture, peritrochanteric fracture, operative time, postoperative complication

## Abstract

Introduction

The high prevalence of obesity among adults in the United States presents significant challenges to orthopedic surgeons. Obesity has been shown to increase operative time and complications in both elective and nonelective orthopedic surgeries. Despite this, there is a lack of literature evaluating the effect obesity has on operative time and postoperative complications following peritrochanteric fracture surgery.

Methods and Materials

We performed a retrospective review of patients who underwent isolated operative stabilization of a peritrochanteric femur fracture at our urban level one trauma center between 2010 and 2018. Patients were divided into an obese group, as defined by a body mass index (BMI) equal to or greater than 30 kg/m^2^,^ ^and a nonobese group, as defined by a BMI less than 30 kg/m^2^. Operative timing variables including time to surgery, operative time, total operating room (OR) time, anesthesia time, and fluoroscopy time were collected. Postoperative variables evaluated including the presence of a major postoperative complication within 90 days of surgery, the need for repeat surgery within 90 days, and the need for surgery due to an infection within 90 days were collected.

Results

A total of 175 patients were included in this retrospective review. Thirty-seven patients were included in the obese group, and 138 were included in the nonobese group. Obesity was associated with a significantly (p = 0.002) longer operative time, total OR time (p = 0.0001), anesthesia time (p = 0.00006), and fluoroscopy time (p = 0.0001). There was no significant difference (p > 0.05) in postoperative variables between the obese and nonobese group. The 90-day major postoperative complication rate was 10.8% in the obese group and 10.9% in the nonobese group. Both repeat surgery and surgery for infection within 90 days were 2.7% in the obese group and 1.4% in the nonobese group.

Conclusion

The treatment of peritrochanteric femur fractures in obese patients is associated with a significantly longer operative time, total OR time, anesthesia time, and fluoroscopy time, but no difference in major postoperative complications when compared to nonobese patients.

## Introduction

Obesity presents significant healthcare challenges throughout all fields of medicine, including orthopedics. It is estimated that the prevalence of obesity among adults in the United States was 42.4% in 2017-2018 [1]. Recent orthopedic literature has shown that obesity can lead to increased complications as well as longer operative times in both elective and nonelective orthopedic surgeries [2-6]. Like obesity, the aging population in the United States has also increased healthcare demand. Each year in the United States, over 300,000 people over the age of 65 are hospitalized for hip fractures [7]. Peritrochanteric fractures, defined as those occurring in the intertrochanteric or subtrochanteric region of the proximal femur, account for a large proportion of hip fractures. Treating peritrochanteric fractures in obese patients presents several potential challenges that must be considered by the patient as well as all providers involved in the patient’s care.

A recent study found obesity to be associated with higher perioperative complication rates and longer operative times when treating intertrochanteric femur fractures [8]. In contrast, the concept of the “obesity paradox” suggests that a high body mass index (BMI) is positively associated with survival following a hip fracture. The “obesity paradox” has been validated in several recent studies looking at post-hip fracture surgery morbidity and mortality [9,10]. Despite this, there remains a paucity of literature investigating the effect obesity has on operative time and postoperative complications following peritrochanteric fracture surgery.

The primary purpose of this study was to evaluate differences in operative times between obese and nonobese patients undergoing operative fixation of peritrochanteric femur fractures. Secondary goals of this study are to compare perioperative timing and postoperative complications within 90 days between obese and nonobese patients undergoing operative fixation of peritrochanteric femur fractures.

## Materials and methods

**Figure 1:**
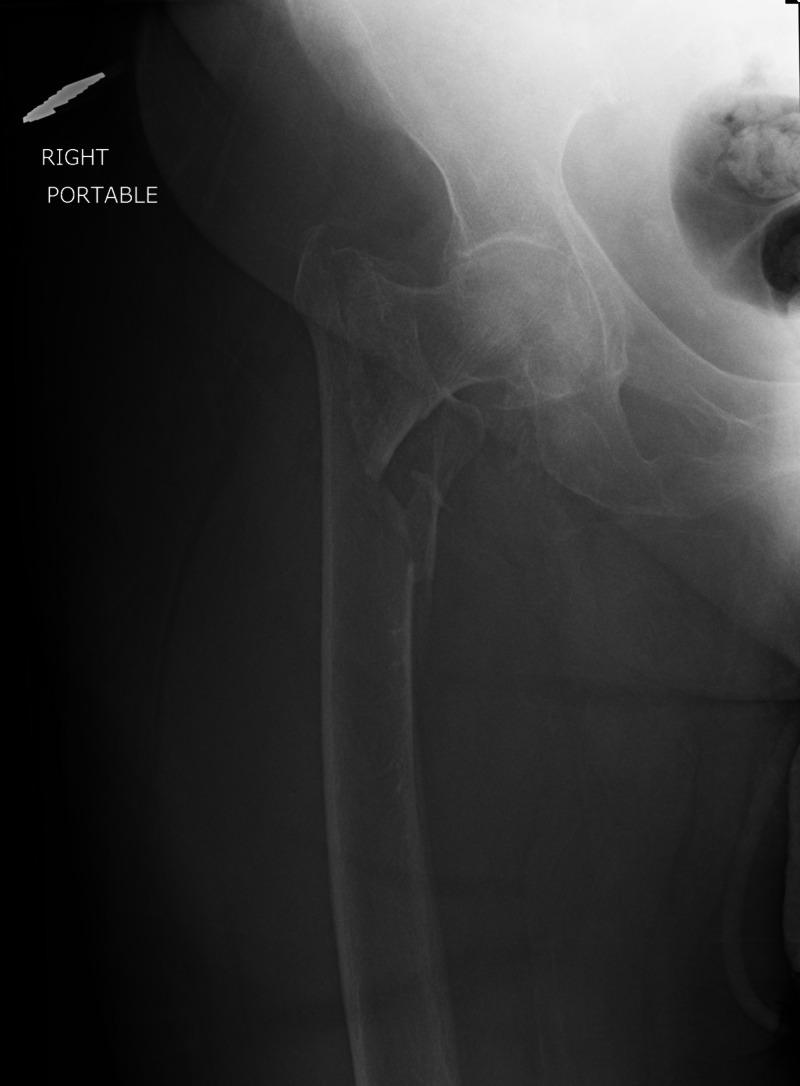
Preoperative Radiograph An anteroposterior radiograph of the right hip demonstrating a displaced intertrochanteric femur fracture with subtrochanteric extension in a patient with a BMI of 31 kg/m^2^

After formal institutional review board approval, we retrospectively reviewed the charts of 175 patients who underwent operative stabilization of a peritrochanteric femur fracture (Figure 1) at our urban level one trauma center between October 1, 2010 and October 31, 2018. Peritrochanteric fractures were defined as those occurring in the intertrochanteric or subtrochanteric region of the proximal femur. Prior to the initiation of this study, an a priori power analysis was performed in order to estimate sufficient sample size needed to achieve adequate power to identify differences in operative time.

**Figure 2:**
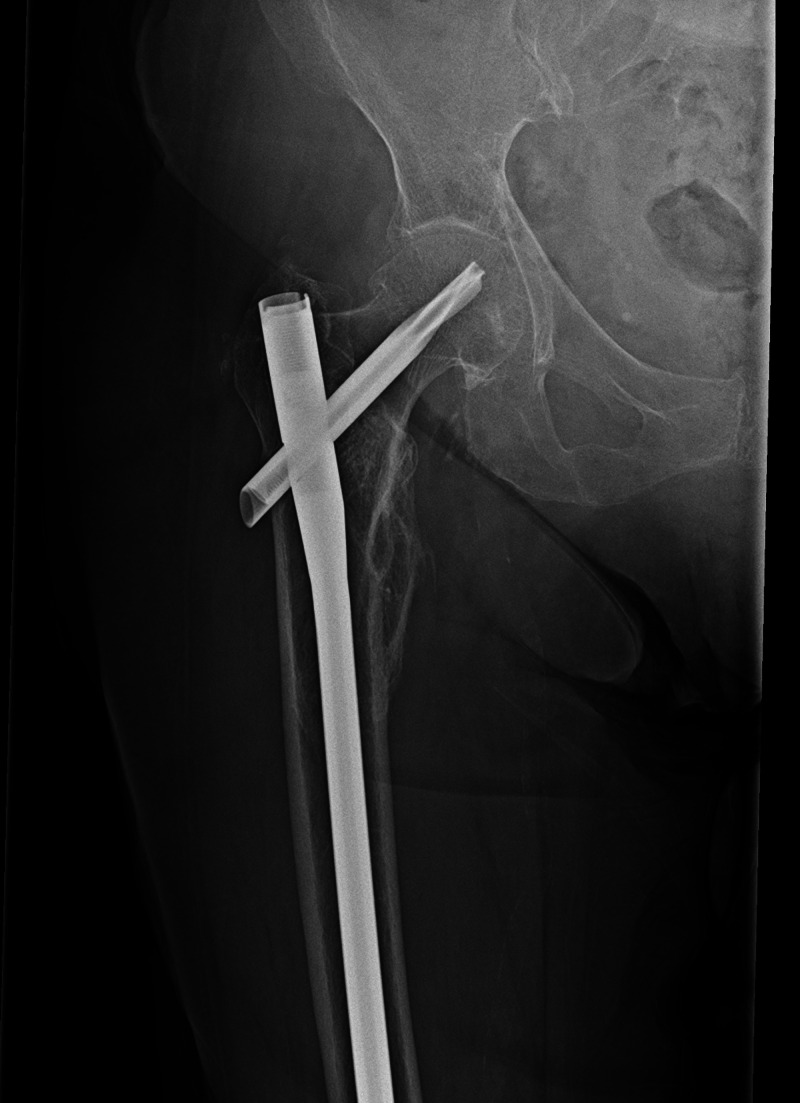
Postoperative Radiograph An anteroposterior radiograph of the right hip one-year status post cephalomedullary nail for the fracture in Figure 1.

The inclusion criteria were patient age equal to or greater than 18 years and patients undergoing an isolated operative procedure for a peritrochanteric femur fracture, including either cephalomedullary nailing (Figure 2) or open reduction and internal fixation (ORIF). Patients were excluded if they had less than three months of follow-up, had previous surgical treatment about the femur, or had multiple operative procedures performed under the same anesthesia episode.

Patients were divided into an obese group, as defined by a BMI equal to or greater than 30 kg/m^2^ and a nonobese group, as defined by a BMI less than 30 kg/m^2^. Demographic data including age, sex, BMI, American Society of Anesthesiologists (ASA) Physical Status, presence of diabetes mellitus, and smoking status were collected. Operative variables collected included time to surgery, operative time, total operating room (OR) time, anesthesia time, and fluoroscopy time. Postoperative variables including the presence of a major postoperative complication within 90 days of surgery, the need for repeat surgery within 90 days, and the need for surgery due to an infection within 90 days were collected. A major postoperative complication was defined as a myocardial infarction, stroke, deep vein thrombosis, pulmonary embolism, deep wound infection, or death. Additional surgeries within 90 days were defined as surgeries related to infection, hardware failure, or a new fracture about the previous operative site.

Statistical analysis

Statistical analysis was performed using Microsoft Excel (Redmond, WA). Data were collected and expressed as means, standard deviations, and percentages. Continuous variables were compared using student’s t-test. Categorical variables were compared using chi-square test. An alpha-level of 0.05 was used to determine statistical significance.

## Results

A total of 175 patients were included in this retrospective review (Table 1). Thirty-seven patients were included in the obese group, and 138 were included in the nonobese group. The obese group was comprised of significantly (p = 0.02) more females (72.9% versus 52%) and significantly (p = 0.005) more diabetics (48.6% versus 27.1%), while the nonobese group had significantly (p = 0.007) more smokers (34% versus 13.5%). The average BMI of the obese group was 35.6 kg/m^2^, while the average BMI of the nonobese group was 23.3 kg/m^2^.

**Table 1:** Demographic Variables Noncategorical variables are expressed as mean ± standard deviation. Categorical variables are given as absolute numbers with percentages in parentheses. ASA, American Society of Anesthesiologists.

Variable	Obese Group	Nonobese Group	P Value
Patients (number)	37	138	-
Age (years)	62 ± 16.9	67.5 ± 20.8	0.14
Sex (female)	27 (72.9%)	72 (52%)	0.02
Body Mass Index (kg/m^2^)	35.6 ± 4.9	23.3 ± 3.4	<0.00001
ASA Physical Status	3 ± 0.64	3.1 ± 0.75	0.43
Diabetic (yes)	18 (48.6%)	34 (27.1%)	0.005
Smoker (yes)	5 (13.5%)	51 (34%)	0.007

Operative variables are presented in Table 2. There was no significant difference in time to surgery between the obese and nonobese groups. Obesity was associated with a significantly (p = 0.002) longer operative time, with the obese group having an average operative time of 73.2 minutes compared to 59.2 minutes in the nonobese group. Analysis of other operative variables demonstrates significantly longer total OR time (p = 0.0001), anesthesia time (p = 0.00006), and fluoroscopy time (p = 0.0001) in the obese group.

**Table 2:** Operative Variables Variables are expressed as mean ± standard deviation.

Variable	Obese Group	Nonobese Group	P Value
Time to surgery (hours)	20.7 ± 13.5	24.4 ± 28.1	0.44
Operative time (minutes)	73.2 ± 25.4	59.2 ± 24.6	0.002
Total operating room time (minutes)	128.3 ± 23.7	110.6 ± 24.4	0.0001
Anesthesia time (minutes)	133.1 ± 24.5	114.1 ± 25.1	0.00006
Fluoroscopy time (minutes)	2.7 ± 1.3	1.9 ± 1.0	0.0001

Table 3 presents the outcomes of postoperative variables. Between the obese and nonobese groups, there was no significant difference in major postoperative complications within 90 days, repeat surgery within 90 days, or surgery for an infection within 90 days. The 90-day major postoperative complication rate was 10.8% in the obese group and 10.9% in the nonobese group. Both repeat surgery and surgery for infection within 90 days were 2.7% in the obese group and 1.4% in the nonobese group.

**Table 3:** Postoperative Variables Categorical variables are given as absolute numbers with percentages in parentheses.

Variable	Obese Group	Nonobese Group	P Value
Major postoperative complication within 90 days (yes)	4 (10.8%)	15 (10.9%)	0.99
Repeat surgery within 90 days (yes)	1 (2.7%)	2 (1.4%)	0.6
Surgery for infection within 90 days (yes)	1 (2.7%)	2 (1.4%)	0.6

## Discussion

This study is one of the few reported series comparing perioperative timing as well as postoperative complications between obese and nonobese patients who underwent operative stabilization of peritrochanteric femur fractures. We found obesity to be associated with a significantly longer operative time; however, there was no difference in major postoperative complications, repeat surgery, or surgery for an infection within 90 days postoperatively when comparing obese to nonobese patients undergoing operative fixation of peritrochanteric fractures. Additionally, we found the obese patients to have significantly longer total OR time, anesthesia time, and fluoroscopy time.

In our study, operative time, total OR time, anesthesia time, and fluoroscopy time were found to be significantly longer in the obese group than in the nonobese group. Previous studies have found obesity to be associated with longer operative times when treating hip fractures [8,11]. In a multicenter retrospective study investigating the effect of obesity on perioperative complications in those undergoing surgical treatment of intertrochanteric femur fractures, Kempegowda et al. found operative time to be statistically longer in obese (96 minutes) versus nonobese (86 minutes) patients [8]. Similarly, Akinleye et al. evaluated patients undergoing a variety of operative treatments for hip fractures and found mean operative time to have a linear relationship with increasing BMI [11]. It is likely that this increased operative time, as well as total OR time found in our study, is due to a multitude of factors. These factors likely include increased difficulty with patient positioning and intraoperative imaging as well as increased difficulty with fracture reduction that may necessitate more frequent need for open reduction. Our study and others demonstrate that operative treatment of hip fractures in obese patients takes significantly more time and work than nonobese patients.

The American Medical Association’s Current Procedural Terminology (CPT) system standardizes healthcare services and procedures and assigns value to them based on the amount of physician time, skill, work, and risk involved. The 22-modifier allows for increased reimbursement in cases when a procedure requires work that is considered more challenging than typical for the procedure being performed [12]. It has recently been reported that the use of the 22-modifier in total hip arthroplasty resulted in significantly higher reimbursement when used in obese patients [13]. Based on our findings, we believe that the use of the 22-modifier for peritrochanteric fracture surgery in obese patients is appropriate. However, further studies evaluating the financial implications of the 22-modifier for peritrochanteric hip fracture surgery is needed, as our study does not address this.

We found no significant difference between obese and nonobese patients in postoperative complications, infection, or need for repeat surgery within 90 days. In a study looking at postoperative medical complications following hip fracture surgery, Batsis et al. found that BMI had no significant influence on postoperative non-cardiac medical complications [14]. In contrast, Kepegowda et al. found obesity to be significantly associated with increased overall postoperative complications (43% vs. 28%, p < 0.0001) in the treatment of intertrochanteric femur fractures [8]. Unlike in total joint arthroplasty, where obesity has been clearly defined as a risk factor for postoperative complications, it appears the relationship between BMI and postoperative complications for hip fracture surgery remains ill-defined [15-17].

As with any study, several inherent weaknesses are present. First, the follow-up period in this study is only 90 days. It is possible that not all major postoperative outcomes would occur in this 90-day period, and therefore our study may underestimate the true postoperative complication rates. Next, the effect of the specific surgical procedure was not evaluated in this study; it is possible that outcomes may be altered if patients undergoing only intramedullary nailing or only ORIF were evaluated. Lastly, the retrospective nature of this study carries an inherent bias that would be improved with prospective data collection.

## Conclusions

Our study shows that the operative treatment of peritrochanteric femur fractures in obese patients is associated with a significantly longer operative time, total OR time, anesthesia time, and fluoroscopy time. However, our study does not demonstrate a significant difference in major postoperative complications when comparing obese and nonobese patients undergoing operative treatment of peritrochanteric femur fractures. Orthopedic surgeons should plan for increased operative time for obese patients with peritrochanteric femur fractures. Lastly, further investigations are necessary to evaluate the effect obesity has on postoperative complications following operative treatment of peritrochanteric femur fractures.
